# Spatial patterns in prostate Cancer-specific mortality in Pennsylvania using Pennsylvania Cancer registry data, 2004–2014

**DOI:** 10.1186/s12885-020-06902-5

**Published:** 2020-05-06

**Authors:** Ming Wang, Emily Wasserman, Nathaniel Geyer, Rachel M. Carroll, Shanshan Zhao, Lijun Zhang, Raymond Hohl, Eugene J. Lengerich, Alicia C. McDonald

**Affiliations:** 1 Department of Public Health Sciences, Penn State College of Medicine and Cancer Institute, 90 Hope Drive, Hershey, PA 17033 USA; 2 Penn State Cancer Institute, Hershey, PA USA; 3 Department of Mathematics and Statistics, the University of North Carolina at Wilmington, Wilmington, NC USA; 4 Biostatistics and Computational Biology Branch, National Institute of Environmental Health Sciences, Research Triangle Park, NC USA; 5 Penn State Institute of Personalized Medicine, Hershey, PA USA; 6 Penn State Milton S. Hershey Medical Center, Hershey, PA USA; 7 Department of Pharmacology, Penn State College of Medicine, Hershey, PA USA

**Keywords:** Prostate cancer, Mortality, Spatial heterogeneity, Catchment area, Accelerated failure time models

## Abstract

**Background:**

Spatial heterogeneity of prostate cancer-specific mortality in Pennsylvania remains unclear. We utilized advanced geospatial survival regressions to examine spatial variation of prostate cancer-specific mortality in PA and evaluate potential effects of individual- and county-level risk factors.

**Methods:**

Prostate cancer cases, aged ≥40 years, were identified in the 2004–2014 Pennsylvania Cancer Registry. The 2018 County Health Rankings data and the 2014 U.S. Environmental Protection Agency’s Environmental Quality Index were used to extract county-level data. The accelerated failure time models with spatial frailties for geographical correlations were used to assess prostate cancer-specific mortality rates for Pennsylvania and by the Penn State Cancer Institute (PSCI) 28-county catchment area. Secondary assessment based on estimated spatial frailties was conducted to identify potential health and environmental risk factors for mortality.

**Results:**

There were 94,274 cases included. The 5-year survival rate in PA was 82% (95% confidence interval, CI: 81.1–82.8%), with the catchment area having a lower survival rate 81% (95% CI: 79.5–82.6%) compared to the non-catchment area rate of 82.3% (95% CI: 81.4–83.2%). Black men, uninsured, more aggressive prostate cancer, rural and urban Appalachia, positive lymph nodes, and no definitive treatment were associated with lower survival. Several county-level health (i.e., poor physical activity) and environmental factors in air and land (i.e., defoliate chemical applied) were associated with higher mortality rates.

**Conclusions:**

Spatial variations in prostate cancer-specific mortality rates exist in Pennsylvania with a higher risk in the PSCI’s catchment area, in particular, rural-Appalachia. County-level health and environmental factors may contribute to spatial heterogeneity in prostate cancer-specific mortality.

## Background

Prostate cancer (PC) is the most common non-skin cancer among U.S. men. Based upon the American Cancer Society’s estimates for year 2019, there are about 174,650 new PC cases in the U.S. PC can be a serious condition contributing to the second leading cause of cancer death in U.S. men after lung cancer, due to the fact that men may progress to more aggressive stages of disease leading to metastasis or death [1]. National forecasts project metastatic PC incidence to increase by 1.03% per year through 2025, with men aged 45–54 years (2.29% per year) and 55–59 years (1.53% per year) increasing more rapidly [2]. Also, in the U.S., it is estimated that about 1 in 41 men will die of PC, and by 2019, about 31,620 deaths due to PC. Even though the five-year survival rate of PC is high (up to 100% if diagnosed at early stage), the diagnosis is likely to be missed at the early stages. In Pennsylvania (PA), 17% of men diagnosed with PC receive their diagnosis after the cancer has spread outside of the prostate [3], which was higher than the national late-stage estimate of 7.9% in 2015. When accounting for overall PC mortality regardless of cancer stage, PC mortality was lower in 2015, from 8.7% compared to 18.9% in the U.S. However, the late-stage PC mortality rates in PA remained high, generally accepted to have a five-year relative survival rate of 28%, as compared to 98% if treated locally [4]. Therefore, it is crucial to recognize high-risk populations and to identify potential risk factors, including spatial heterogeneity that may be associated with PC-related mortality in PA.

**Fig. 1:**
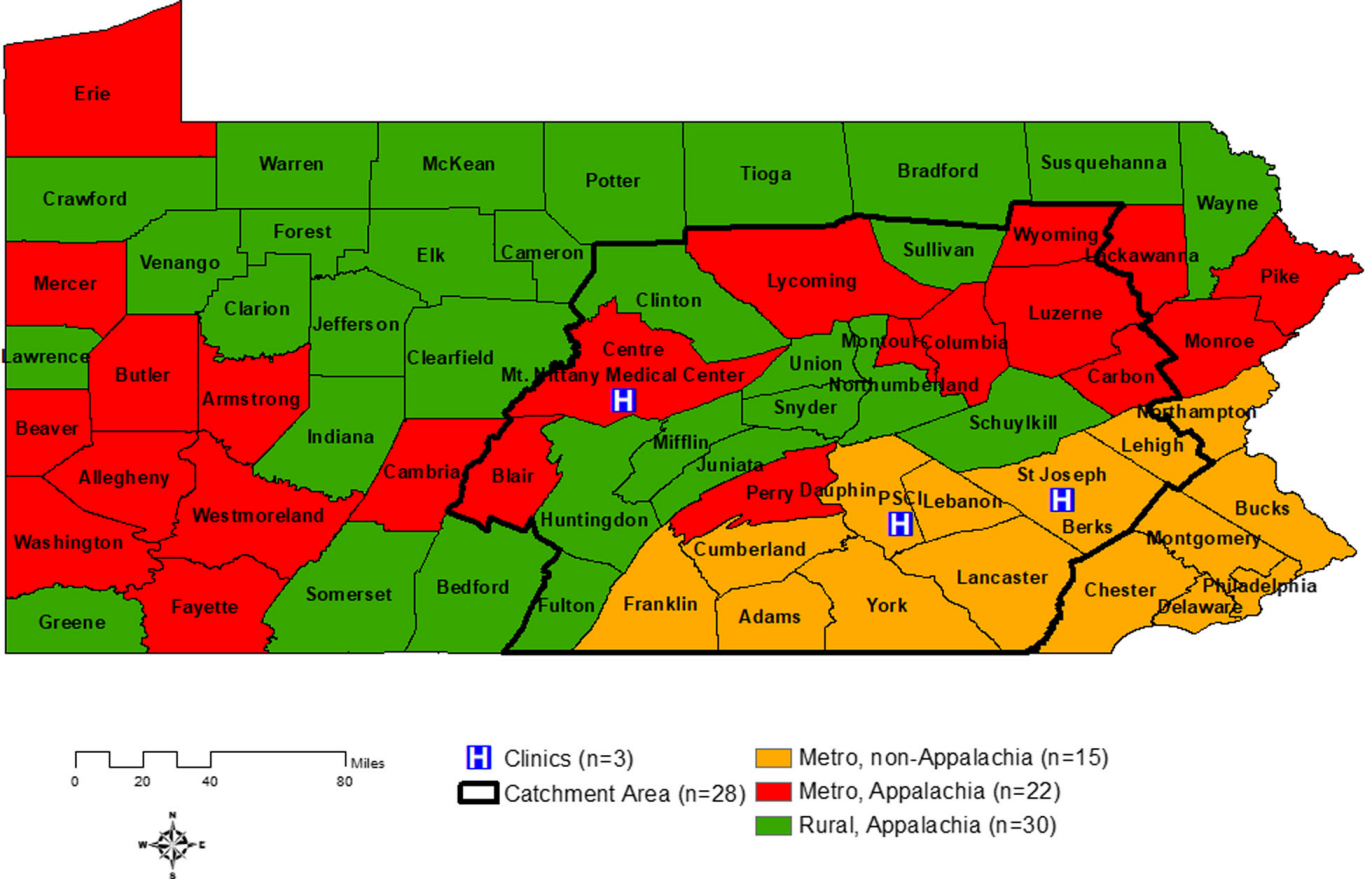
Map of Pennsylvania by the Urban or Rural Appalachia regions and the PSCI Catchment Area. In Pennsylvania, there are 15 counties in the Metro (Urban), non-Appalachian region, 22 counties in the Metro (Urban), Appalachian region, and 30 counties in the Rural, Appalachian region. In particular, the PSCI Catchment area includes 28 counties (within a black boarded line) in Central Pennsylvania, with 9 Metro (Urban), non-Appalachian counties, 9 Metro (Urban), Appalachian counties and 10 Rural, Appalachian counties

According to the North American Association of Central Cancer Registries (including the U.S. and Canada), rural areas have significantly higher incidence rates of PC compared to other geographical areas [5]. Despite this geographical disparity, the PC burden in PA, with nearly half the region occupied by rural areas (30 rural counties among 67 counties), in particular, in Central PA, is increasing due to several potential factors, such as relatively high numbers of Hispanics migrating to rural or non-metropolitan areas [6]. The Penn State Cancer Institute (PSCI) headquartered at the Milton S. Hershey Medical Center (Dauphin County), part of Penn State Health, is the only academic cancer center in central PA with primary and specialty care. Its catchment area consists of 28 counties, 10 rural (non-metro) Appalachia, 9 urban (metro) Appalachia, and 9 urban (metro) non-Appalachia areas, with a three-hour driving distance (approximately 160-mile radius) to the cancer center; with an estimated 4 million residents (33% of PA population) (Fig. 1). The PSCI’s goals are to investigate factors for cancer risk and poor cancer outcomes and to reduce these risks and improve cancer health outcomes in (central) PA. In order to accomplish these goals for PC, PC risk and outcomes need to be fully understood in this area. However, few studies have investigated PC-specific mortality and its spatial heterogeneity in this area.

To better understand the risk and spatial pattern of PC mortality in PA, it is crucial to identify potentially associated risk factors for PC mortality. Several well-known factors for PC have been established in the literature [7, 8]. For instance, black men have been identified to have a higher risk for PC and are about 2.5 times more likely to die from PC compared to non-Hispanic white men [9, 10]. One possible reason is racial disparity regarding cancer treatment in PA, which could impact quality of healthcare and physician-patient communications [11, 12]. Other contributing factors include limited access to healthcare and PC screening, low socioeconomic status, environmental or occupational exposure to heavy metals, participation in unhealthy lifestyle behaviors (i.e., cigarette smoking and lack of physical activity), and variation in cancer beliefs and perceptions [13–16]. Therefore, it is recognized that analyzing PC-specific mortality is a multifactorial process that involves the assessment of interactions amongst patients, providers and healthcare facilities, and their communities. In practice, a lot of influential factors may be unknown or inaccessible for quantification due to the complexity, high cost, feasibility or ethical impermissibility (e.g. private socio-economic factors or genomic data from cancer tumor) [17]. Furthermore, these factors could vary substantially across geographic locations [18]. Thus, the consideration of geospatial variation is of utmost importance when evaluating the characteristics of a cancer center’s catchment area, such as the proximity and dependency among adjacent counties in relation to certain risk factors and cancer outcomes [17]. The county at the time of diagnosis can be used as a surrogate measure to capture and link certain geographical information from other external sources, information that is unavailable in the existing database [13]. To achieve these goals, advanced geospatial survival analysis techniques need to be adopted to draw valid inferences.

In this study, we utilized the 2004–2014 Pennsylvania Cancer Registry (PCR) data to examine PC mortality risk in PA with a focus on PSCI’s catchment area considering urban and rural Appalachian regions [19, 20] and potential risk factors that may contribute to PC mortality. We used epidemiological techniques for incidence rate calculations, in addition to applying more advanced spatial statistical approaches. Spatial correlation was incorporated into PC-specific survival analyses, while also accounting for individual-level risk factors. Secondary assessment of county-level risk factors, from the 2018 Pennsylvania County Health Rankings (CHR) Data [21–23] and the 2014 Environmental Protection Agency (EPA) Environmental Quality Index (EQI) [24, 25] based on the estimated spatial frailties from survival models, was explored to evaluate their potential associations with spatial heterogeneity. This investigation allows us to gain a better understanding of PC mortality in PA and the PSCI catchment area, while simultaneously generating hypotheses for future directions in addressing PC-related disparities.

## Methods

### Pennsylvania Cancer registry (PCR) study population

From the population-based PCR between 2004 and 2014, we included men, aged ≥40 years, who had a primary, clinical diagnosis of PC with a Gleason score [GS] ≥ 6. PC cases who had a missing GS were also included if the tumor stage was T3 or T4. PC cases were classified into the following disease groups (PC aggressiveness): 1) less aggressive PC (GS 6 or 7 (3 + 4) **and** the tumor stage was T1-T2 **and** no distant metastasis) and 2) more aggressive PC (GS ≥ 7 (4 + 3) **or** a tumor stage T3-T4 **or** distant metastasis). Note that for PC cases with both a documented pathology GS (at surgery, prostatectomy, or autopsy) and a clinical GS (at biopsy, TURP), the pathology GS was used. The pathology tumor stage was used for cases where a clinical tumor stage was also documented. If pathology GS or pathology tumor stage was not available, clinical GS and clinical tumor stage was used, respectively.

PC-specific deaths were based on the ICD-O-2/3 primary site code (C61, C619) that were extracted from the PCR; and, deaths due to other causes were treated as censored data. The urban or rural Appalachia status for each PC case was determined by their county of residence at the time of diagnosis based on the Appalachian Regional Commission definition [19] and the U.S. Department of Agriculture of Rural-Urban Continuum Codes (RUCC) definition (RUCC< 4 for metro [urban] areas; others for non-metro [rural] areas) [20]. Figure 1 shows the 28 counties located in the PSCI catchment area and the other PA counties in the non-catchment area. Informed consent of PC cases was waived; and, the study was approved by the Institutional Review Boards (IRBs) of the Pennsylvania Department of Health and the Pennsylvania State University College of Medicine.

### County health rankings (CHR) data and environmental quality index (EQI) data

To identify other potential factors associated with spatial variations in PC-specific mortality, all county-level data were extracted from the 2018 CHR and the 2014 EQI data, which are most recent available resources. The CHR data includes health behaviors, clinical care, sociology, economics, and physical environment indicators (*http://www.countyhealthrankings.org**).* The EQI data consist of air, water, land, built, and socio-demographic domains that provide information on the overall quality of the environment in the U.S. (*https://edg.epa.gov**).* From both databases, a total of 270 variables were extracted for the secondary assessment analysis.

### Statistical analysis

Summary statistics are presented by means with standard deviations (SD) for continuous variables and frequencies with percentages for categorical variables. To assess the differences of demographic and clinical characteristics between geographical regions by the PSCI catchment area, and also between urban and rural Appalachian regions within the PSCI catchment area, one-way ANOVA and Chi-square tests were applied. Age-adjusted incidence rates (per 100,000 men) for PC and more aggressive PC were calculated based on the 2000 US Standard Population, with standard population weights corrected for a subpopulation aged 40 and above. The 95% confidence intervals were obtained using the Gamma method [26].

For PC-specific survival, Kaplan-Meier estimates stratified by geographical regions were calculated; and, group comparisons were based on log-rank tests. Here, we adopted multivariable accelerated failure time (AFT) models to investigate the association between survival and various risk factors, with a spatial frailty term accounting for spatial correlation and representing geographical variation [27–29]. The individual-level risk factors such as age at diagnosis, race, ethnicity, insurance status, aggressiveness, lymph nodes, treatment received, and geographical regions at the time of diagnosis (i.e., urban or rural Appalachia, the PSCI catchment and non-catchment areas) were obtained from the PCR, and were initially screened based on univariate analyses, prior knowledge in literature and primary associations of interest before being considered for multivariate AFT models. The Bayesian estimates with 95% credible intervals are reported. Furthermore, we performed univariate secondary assessment on the CHR and EQI data by accounting for the spatial structure to identify other potential health-related or environmental factors, which may contribute to PC mortality. All the parameter estimation and inference were conducted under the Bayesian framework, and the models were evaluated based on goodness of fit using the deviance inference criterion. GIS mapping was used to show the distribution of Urban or Rural Appalachian regions in PA, and also the spatial variation of PC-specific survival based on the estimated spatial frailties from the AFT model fitting.

All analyses were conducted in software R (version 3.5.1). The standardized age-adjusted incidence analysis was performed by the R package dsr. For the AFT model fitting [30], the R package R2WinBUGS was adopted by calling the Bayesian computing software WinBUGS [31, 32]. All tests were two-sided with the significance level of 0.05. All maps were generated in ArcGIS (version 10.6.1).

## Results

There were 102,194 PC cases in men from the PCR diagnosed between 2004 and 2014. Based on our inclusion and exclusion criteria, there were a total of 7920 PC cases excluded due to a GS < 6 (*n* = 2094), or a missing GS but without the tumor stage of T3 or T4 (*n* = 5768), or had a missing age or did not meet the age criteria of ≥40 years (*n* = 58). There were 94,274 cases eligible for analysis. Of the eligible cases, 56,121 men had less aggressive PC (15,822 in catchment area, 28.2%) and 30,931 men had more aggressive PC (9078 in catchment area, 29.3%). As shown in Table 1, the majority (83.9%) of the cases were of white race in PA with a larger proportion in the catchment area (92.4%) compared to the non-catchment area (80.4%, i.e. the remainder of PA). Compared to the non-catchment area, the catchment area cases were older in age at the time of diagnosis, had a higher serum PSA, were less likely to be insured, had a higher proportion with a GS of 8–10, were less likely to have positive lymph nodes (LN) and were less likely to receive definitive treatment. Within the catchment area, rural Appalachian cases were older in age, less likely to be insured, more likely to have positive LN, more likely to have distant metastasis, and were less likely to receive definitive treatment compared to urban Appalachia and urban Non-Appalachia. Cases from urban Appalachia in the catchment area had a higher serum PSA on average and a larger proportion of GS 8–10 compared to rural Appalachia and urban non-Appalachia cases diagnosed in the catchment area.

**Table 1:** Descriptive Characteristics of Eligible Prostate Cancer Cases Diagnosed at Age 40+ in PCR, 2004–2014

Variables	PA (***N*** = 94,274)	PA		Catchment Area		
		Catchment Area (***N*** = 27,357, 29.02%)	Non-Catchment Area (***N*** = 66,917, 70.98%)	Rural Appalachia (***N*** = 3828, 13.99%)	Urban Appalachia (***N*** = 6483, 23.70%)	Urban Non-Appalachia (***N*** = 17,046, 62.31%)
**Mean Age** in years (SD)^a,b^	66.38 (9.38)	66.73 (9.37)	66.24 (9.38)	66.95 (9.31)	66.47 (9.31)	66.77 (9.40)
**Mean Serum PSA,** ng/mL (SD) ^a,b^	12.21 (19.03)	12.43 (18.88)	12.12 (19.09)	12.97 (19.83)	13.11 (19.73)	12.04 (18.3)
*Missing #*	11,963	3667	8296	583	714	2370
**Race***n (%)*^a,b^						
White	79,066 (83.87)	25,278 (92.40)	53,788 (80.38)	3681 (96.16)	6171 (95.19)	15,426 (90.50)
Black	10,067 (10.68)	1038 (3.79)	9029 (13.49)	64 (1.67)	126 (1.94)	848 (4.97)
Asian	571 (0.61)	91 (0.33)	480 (0.72)	3 (0.08)	16 (0.25)	72 (0.42)
Other/Unknown	4570 (4.85)	950 (3.47)	3620 (5.41)	80 (2.09)	170 (2.62)	700 (4.11)
**Ethnicity***n (%)*^a,b^						
Hispanic	1309 (1.39)	528 (1.93)	781 (1.17)	25 (0.65)	60 (0.93)	443 (2.60)
Non-Hispanic	80,271 (85.15)	23,411 (85.58)	56,860 (84.97)	3057 (79.86)	5230 (80.67)	15,124 (88.72)
Unknown	12,694 (13.47)	3418 (12.49)	9276 (13.86)	746 (19.49)	1193 (18.40)	1479 (8.68)
**Insurance***n (%)*^a,b^						
Insured	76,737 (81.40)	22,045 (80.58)	54,692 (81.73)	3049 (79.65)	5296 (81.69)	13,700 (80.37)
Uninsured	443 (0.47)	161 (0.59)	282 (0.42)	24 (0.63)	18 (0.28)	119 (0.70)
Unknown	17,094 (18.13)	5151 (18.83)	11,943 (17.85)	755 (19.72)	1169 (18.03)	3227 (18.93)
**Gleason Score***n (%)*^a,b^						
6	40,762 (43.24)	11,768 (43.02)	28,994 (43.33)	1754 (45.82)	2762 (42.60)	7252 (42.54)
7	37,531 (39.81)	10,725 (39.20)	26,806 (40.06)	1398 (36.52)	2550 (39.33)	6777 (39.76)
8–10	15,525 (16.47)	4723 (17.26)	10,802 (16.14)	655 (17.11)	1140 (17.58)	2928 (17.18)
Unknown	456 (0.48)	141 (0.52)	315 (0.47)	21 (0.55)	31 (0.48)	89 (0.52)
**Tumor Stage***n (%)*^b^						
T1	37,957 (40.26)	11,118 (40.64)	26,839 (40.11)	1617 (42.24)	2532 (39.06)	6969 (40.88)
T2	41,341 (43.85)	11,851 (43.32)	29,490 (44.07)	1639 (42.82)	2779 (42.87)	7433 (43.61)
T3	8539 (9.06)	2477 (9.05)	6062 (9.06)	290 (7.58)	607 (9.36)	1580 (9.27)
T4	1338 (1.42)	391 (1.43)	947 (1.42)	62 (1.62)	101 (1.56)	228 (1.34)
Unknown	5099 (5.41)	1520 (5.56)	3579 (5.35)	220 (5.75)	464 (7.16)	836 (4.90)
**Distant Metastasis***n (%)*^b^						
Yes	3580 (3.80)	1028 (3.76)	2552 (3.81)	165 (4.31)	269 (4.15)	594 (3.48)
No	82,355 (87.36)	23,468 (85.78)	58,887 (88.00)	3141 (82.05)	5418 (83.57)	14,909 (87.46)
Unknown	8339 (8.85)	2861 (10.46)	5478 (8.19)	522 (13.64)	796 (12.28)	1543 (9.05)
**Aggressiveness***n (%)*^a,b^						
Less Aggressive	56,121 (59.53)	15,822 (57.84)	40,299 (60.22)	2217 (57.92)	3646 (56.24)	9959 (58.42)
More Aggressive	30,931 (32.81)	9078 (33.18)	21,853 (32.66)	1174 (30.67)	2177 (33.58)	5727 (33.60)
Unknown	7222 (7.66)	2457 (8.98)	4765 (7.12)	437 (11.42)	660 (10.18)	1360 (7.98)
**Lymph Node Positive***n (%)*						
No	81,875 (86.85)	23,458 (85.75)	58,417 (87.30)	3153 (82.37)	5474 (84.44)	14,831 (87.01)
Yes	2327 (2.47)	642 (2.35)	1685 (2.52)	93 (2.43)	151 (2.33)	398 (2.33)
Unknown	10,072 (10.68)	3257 (11.91)	6815 (10.18)	582 (15.20)	858 (13.23)	1817 (10.66)
**Definitive Treatment Regimen***n (%)*^a,b^						
Radiation Only	35,373 (37.52)	10,259 (37.50)	25,114 (37.53)	1294 (33.80)	2357 (36.36)	6608 (38.77)
Primary Site Surgery Only	31,985 (33.93)	8863 (32.40)	23,122 (34.55)	1070 (27.95)	1954 (30.14)	5839 (34.25)
Both Treatments Received	1976 (2.10)	580 (2.12)	1396 (2.09)	88 (2.30)	139 (2.14)	353 (2.07)
Neither Treatment Received	22,707 (24.09)	6783 (24.79)	15,924 (23.80)	1271 (33.20)	1810 (27.92)	3702 (21.72)
Unknown/Missing	2233 (2.37)	872 (3.19)	1361 (2.03)	105 (2.74)	223 (3.44)	544 (3.19)

Note: 1) Rural refers to Non-Metro (RUCC≥4); Urban refers to Metro (RUCC< 4); 2) All reported percentages are column percentages; 3) PSA = Prostate-Specific Antigen (PCR documentation top-coded at 98.0 and bottom-coded at 0.1); 4) Tumor Stage was based on TNM staging system; 5) Primary Site Surgery refers only to total organ resection (radical prostatectomy not otherwise specified [NOS], total prostatectomy NOS, prostatectomy with resection in continuity with other organs, prostatectomy NOS); 6) Unknown/Missing/Other categories were not included in statistical tests of association; 7) Chi-Square tests are used for categorical characteristics and one-way ANOVA are for continuous characteristics

^a^ Significant difference between the Catchment and Non-Catchment Areas in PA

^b^ Significant associations among Appalachian-RUCC Regions within the Catchment Area

**Fig. 2:**
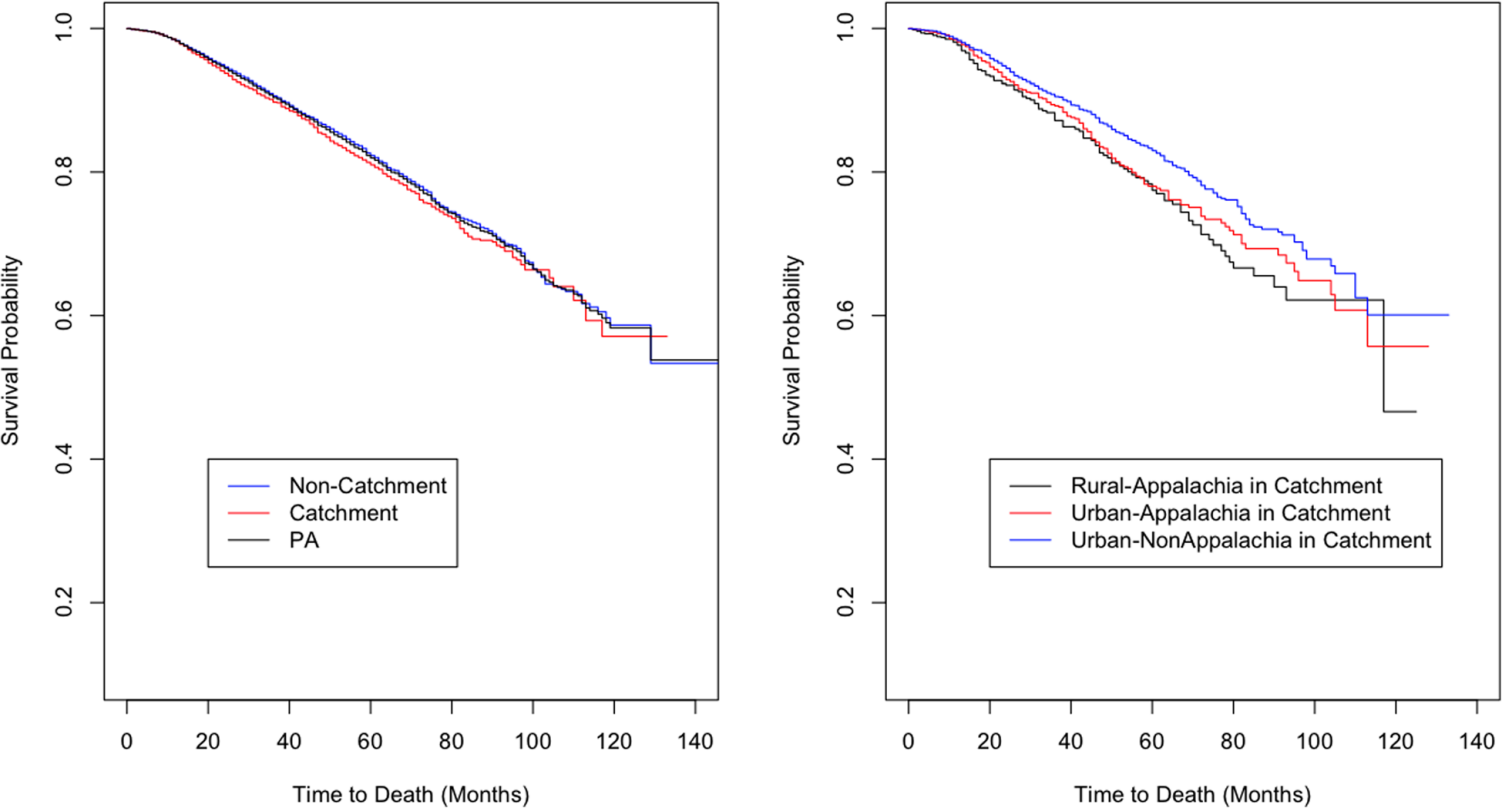
The Kaplan-Meier curves for Prostate Cancer-specific survival by the PSCI catchment area and by the Urban or Rural Appalachia regions within the PSCI catchment area. The Kaplan-Meier curves for Prostate Cancer-specific survival by the PSCI catchment area are not significantly different with *p*-value = 0.1. Also, the Kaplan-Meier curves for Prostate Cancer-specific survival by the Urban or Rural Appalachia Regions within the PSCI catchment area are significantly different with p-value = 0.001. Note that, *p*-values for group comparisons on survival curves are obtained from the log-rank tests

Figure 2 shows that the catchment area had lower survival rates (higher mortality rates) compared to the non-catchment area; however, there was no statistically significant difference detected (*p*-value = 0.1). In rural-Appalachia, the catchment area had a statistically significantly higher risk of mortality compared to the non-catchment area (*p*-value = 0.002, see Supplementary material). Within the PSCI catchment area, rural Appalachia had statistically significantly lower survival rates (higher mortality rates) compared to urban Appalachia and urban non-Appalachia (*p*-value = 0.001), and a similar pattern was observed for PA (see Supplementary material).

Table 2 summarizes PC-specific survival and incidence. Overall for PA, the 2004–2014 incidence rates of PC and more aggressive PC were 276.68 and 92.43 per 100,000 men, respectively. The catchment area had lower 2004–2014 incidence rates of PC and more aggressive PC (249.68 per 100,000 men and 84.63 per 100,000 men, respectively) compared to the non-catchment area (289.56 per 100,000 men and 96.15 per 100,000 men, respectively). Within the catchment area, rural Appalachia had the highest incidence of PC (252.59 per 100,000 men) and urban non-Appalachia had the highest incidence of more aggressive PC (85.88 per 100,000 men). As for PC-specific survival, the 3-, 5-, and 10-year survival rates for overall PA were 90.4, 82.0, and 58.3%, respectively. The catchment area had consistently lower survival rates (89.8, 81, 57.1%, respectively) compared to the non-catchment area (90.6, 82.3, and 58.7%, respectively). Within the catchment area, rural Appalachia had the lowest survival rates (87.2, 77.4, and 46.6%, respectively) and urban non-Appalachia had the highest (90.5, 83.0, 60.1%, respectively).

**Table 2:** Age-adjusted Incidence and PC-Specific Survival Rates (95% Confidence Intervals, CI) for PC Cases diagnosed at age 40+ in the PCR, 2004–2014

Measures	PA	PA		Catchment Area		
		Catchment Area	Non-Catchment Area	Rural Appalachia	Urban Appalachia	Urban Non-Appalachia
***Incidence Rates (95% CI)***						
PC	276.68 (274.90, 278.47)	249.68 (246.69, 252.68)	289.56 (287.35, 291.79)	252.59 (244.58, 260.79)	246.61 (240.58, 252.76)	250.31 (246.52, 254.14)
More Aggressive PC	92.43 (91.39, 93.48)	84.63 (82.88, 86.41)	96.15 (94.86, 97.45)	78.99 (74.50, 83.69)	84.73 (81.17, 88.41)	85.88 (83.65, 88.16)
***Survival Rates (95% CI)***						
3-year	0.904 (0.899, 0.909)	0.898 (0.888, 0.908)	0.906 (0.900, 0.912)	0.872 (0.844, 0.901)	0.892 (0.872, 0.914)	0.905 (0.893, 0.918)
5-year	0.820 (0.811, 0.828)	0.810 (0.795, 0.826)	0.823 (0.814, 0.832)	0.774 (0.732, 0.819)	0.780 (0.746, 0.815)	0.830 (0.811, 0.849)
10-year	0.583 (0.549, 0.619)	0.571 (0.505, 0.645)	0.587 (0.547, 0.629)	0.466 (0.261, 0.832)	0.557 (0.449, 0.691)	0.601 (0.528, 0.684)

Note, *CI* confidence interval

To examine spatial heterogeneity for PC-specific mortality, geospatial AFT models with the spatial frailty term accounting for the geographical variation were fitted for PA and the PSCI catchment area. The individual-level risk factors were screened (see more details in Supplementary material), and included race, ethnicity, insurance status, aggressiveness, lymph nodes, treatment received, rurality-Appalachia and catchment regions for final AFT model fitting. After removing PC cases due to missing data in selected risk factors, there were 63,224 cases included for analysis. Table 3 summarizes the regression results for the fixed effect parameters. Of note, the estimates are directly associated with the natural logarithm of time, with a negative value indicating a decrease in survival time and a positive value for an increase in survival time. For instance, for the catchment area, the average survival time of PC cases who were from rural Appalachia was 20% (1-exp(− 0.221) with 95% credible interval, CI, of 7–31%) less than those from urban non-Appalachia. Also, statistically significantly lower PC-specific survival time was observed for cases who were not insured compared to insured, had more aggressive PC at the time of diagnosis compared to less aggressive PC, and positive LN compared to negative LN. Higher PC-specific survival time was observed for cases with any definitive PC treatment compared to those without either primary site surgery or radiation treatments, using the data solely within the PSCI catchment area. For example, the average survival time of PC cases who received both surgery and radiation was 3.11 (exp(1.134) with 95% CI of 2.19–4.55) times higher than those who did not receive either. In addition, regarding the AFT model fitting for PA, besides similar significant effects of other risk factors, the results also show urban Appalachia having lower PC-specific survival time compared to urban non-Appalachia (reduction percentage of 14% with 95% CI of 7–21%).

**Table 3:** Parameter Estimates from multivariable spatial survival regressions via accelerated failure time models on Prostate Cancer-specific Survival in PA and the PSCI catchment area for PC Cases diagnosed at age 40+ in the PCR, 2004–2014

Variables	PA (*N* = 63,224)	Catchment Area (*N* = 17,732)
	Estimate (95% CI)	Estimate (95% CI)
***Appalachian-RUCC Regions***		
Urban Non-Appalachia	Ref	Ref
Rural Appalachia	***−0.170 (− 0.253, − 0.073)***	***− 0.221 (− 0.368, − 0.076)***
Urban Appalachia	***−0.152 (− 0.232, − 0.077)***	−0.065 (− 0.213, 0.223)
***Race***		
White	Ref	Ref
Black	***−0.103 (− 0.193, − 0.010)***	−0.030 (− 0.311, 0.271)
Asian	***0.426 (0.001, 0.920)***	0.041 (−0.736, 0.932)
***Hispanic***		
No	Ref	Ref
Yes	0.051 (−0.200, 0.330)	0.107 (−0.305, 0.537)
***Insurance***		
Insured	Ref	Ref
Not Insured	***−0.586 (− 0.821, − 0.349)***	***−0.564 (− 1.001, − 0.126)***
***Aggressiveness***		
Less Aggressive	Ref	Ref
More Aggressive	***−1.477 (− 1.545, − 1.412)***	***−1.571 (− 1.700, − 1.462)***
***Lymph Node Positive***		
No	Ref	Ref
Yes	***−0.819 (− 0.895, − 0.738)***	***−0.807 (− 0.941, − 0.659)***
***Definitive Treatment Regimen***		
Neither Treatment Received	Ref	Ref
Primary Site Surgery Only	***1.283 (1.186, 1.396)***	***1.232 (1.026, 1.440)***
Radiation only	***0.602 (0.542, 0.665)***	***0.637 (0.514, 0.768)***
Both Treatments Received	***0.977 (0.812, 1.161)***	***1.134 (0.782, 1.516)***
***Catchment Area***		
No	Ref	–
Yes	***−0.067 (− 0.136, − 0.004)***	–

Note: Unknown/missing data in risk factors are removed before model fitting; CI: credible interval; The estimates in bold indicates statistical significance

**Fig. 3:**
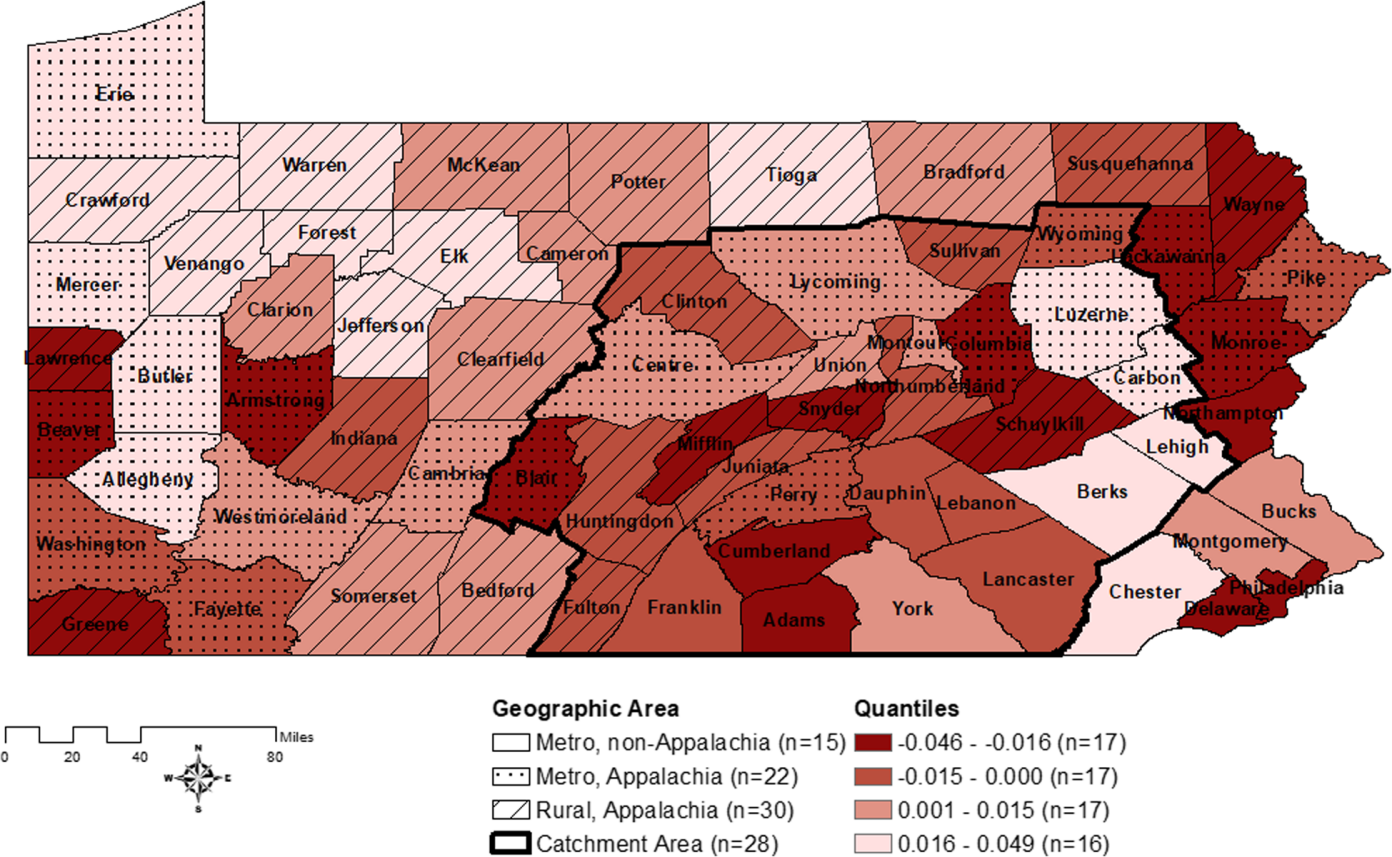
Estimated spatial frailties from the Prostate Cancer-specific AFT model for Pennsylvania. The multivariable Prostate Cancer-specific AFT model is considered with individual-level risk factors, the Urban or Rural Appalachia regions and the PSCI catchment areas for adjustment. The GIS map is displayed based on quantile classification

Figure 3 displays the PA map of the spatial frailty parameter estimates with higher values indicating longer PC-specific survival (darker color indicates lower survival), with the detailed output by county provided in the supplementary material. Counties located in south central PA (majority within the catchment area, i.e., Cumberland, Snyder, Blair, Adams and Mifflin), southwestern PA (i.e., Lawrence, Beaver, Greene, Armstrong) and along the eastern PA border (i.e., Delaware, Philadelphia, Northampton, Wayne, Lackawanna and Monroe) exhibited shorter survival times; and counties located in northwestern PA (i.e., Erie, Crawford, Warren, Venango, Forest, Mercer) and the eastern region of the PSCI Catchment area border (i.e., Berks, Lehigh, Carbon, Luzerne) exhibited longer survival times.

To identify other potential risk factors for the distribution of spatial frailty associated with PC-specific survival (or mortality), secondary assessment on CHR data and environmental factors obtained from the EQI accounting for spatial correlation structure was conducted. The factors with statistically significant differences between the 1st and 4th quartiles of counties based on spatial frailty estimates from the secondary geospatial regression models are listed in Table 4. Descriptive statistics (mean ± SD) of those selected factors, specifically for the 1st and 4th quartiles of counties, are provided in the supplementary material. From the CHR data, counties with a shorter survival time (i.e., higher risk of PC-specific mortality) were reported to have more poor physical health days/physically unhealthy days, higher percentages of low birth weights, higher premature age-adjusted mortality, and a higher prevalence of diabetes. Also, longer survival (lower risk of PC mortality) was associated with higher value of food environment index, median household income, income inequality 80th percentile, and number of workers driving alone/long commute. From the EQI data, several environmental risk factors on land and in the air were identified. In particular, higher herbicides and insecticides but lower percentages of defoliate chemical applied/total acres were associated with lower PC-specific mortality. Furthermore, higher amounts of air emissions in 1,1,2-trichloroethane, 2-nitropropane, acrylic acid, antimony compounds, o-toluidine, bromoform, dimethyl sulfate, and vinyl acetate (among many others listed in Table 4) were associated with higher PC-specific mortality. More details can be referred to in the Supplementary Materials.

**Table 4:** The list of selected environmental factors which are significant for the 4th vs. 1st quartile based on univariate secondary assessment of spatial frailties from the AFT model in PA for PC Cases diagnosed at age 40+ in the PCR, 2004–2014

	Estimate (95% CI)
**County Health Rankings**^b^	
*Poor physical health days/Physically Unhealthy Days (N)*	− 0.62 (− 1.23, − 0.01)
*Low birth weight (%)*	− 0.70 (− 1.29, − 0.11)
*Food Environment Index*	0.61 (0.05, 1.15)
*Income inequality 80th Percentile*	0.51 (0.02, 1.01)
*Number of Workers driving alone/long commute (N)*	0.52 (0.04, 0.99)
*Premature Age-Adjusted Mortality*	−0.60 (−1.17, − 0.02)
*Diabetes prevalence/Diabetic (N)*	− 0.67 (− 1.23, − 0.09)
*Median household income*	0.56 (0.06, 1.05)
**Land Domain**^c^	
*Percent defoliate chemical applied/total acres*^a^	−1.66 (−3.06, − 0.26)
*Herbicides (pounds)*^a^	1.18 (0.24, 2.15)
*Insecticides (pounds)*^a^	0.95 (0.03, 1.89)
**Air Domain**^c^	
*1,1,2-trichloroethane (tons emitted)*^a^	−1.75 (−2.89, −0.65)
*2,4-toluene diisocyanate (tons emitted)*^a^	−1.15 (− 1.98. -0.33)
*2-nitropropane (tons emitted)*^a^	− 1.13 (− 1.90, − 0.42)
*Acetonitrile (tons emitted)*^a^	− 0.94 (− 1.81, − 0.09)
*Acetophenone (tons emitted)*^a^	− 0.81 (− 1.53, − 0.12)
*Acrylic acid (tons emitted)*^a^	−1.64 (− 2.82, − 0.48)
*Antimony compounds (tons emitted)*^a^	−1.08 (− 2.05, − 0.14)
*Benzyl chloride (tons emitted)*^a^	−1.38 (− 2.22, − 0.58)
*Bromoform (tons emitted)*^a^	−2.34 (− 3.56, − 1.16)
*Chloroprene (tons emitted)*^a^	−1.11 (− 1.91, − 0.34)
*Dibutylphthalate (tons emitted)*^a^	−0.96 (− 1.78, − 0.17)
*Dimethyl phthalates (tons emitted)*^a^	−1.20 (− 2.11, − 0.32)
*Dimethyl sulfate (tons emitted)*^a^	−1.53 (− 2.41, − 0.70)
*Epichlorohydrin (tons emitted)*^a^	−1.32 (− 2.20, − 0.49)
*Ethyl acrylate (tons emitted)*^a^	−1.68 (− 2.90, − 0.49)
*Ethylidene dichloride (tons emitted)*^a^	−1.25 (− 2.37, − 0.17)
*Hexachlorobenzene (tons emitted)*^a^	−0.92 (− 1.55, − 0.32)
*Hexachlorobutadiene (tons emitted)*^a^	−0.84 (− 1.49, − 0.25)
*Hydrazine (tons emitted)*^a^	−0.79 (− 1.46, − 0.15)
*Isophorone (tons emitted)*^a^	−1.27 (− 2.34, − 0.23)
*Methylhydrazine (tons emitted)*^a^	−1.14 (− 1.93, − 0.39)
*Nitrobenzene (tons emitted)*^a^	−0.84 (− 1.48, − 0.24)
*N,N-dimethylaniline (tons emitted)*^a^	−0.61 (− 1.23, − 0.02)
*o-toluidine (tons emitted)*^a^	−1.18 (− 1.98, − 0.43)
*Vinyl acetate (tons emitted)*^a^	−0.85 (− 1.72, − 0.08)

^a^ Risk factor was log-transformed;

*CI* credible interval;

^b^https://www.countyhealthrankings.org/explore-health-rankings/measures-data-sources/;

^c^https://edg.epa.gov/data/Public/ORD/NHEERL/

## Discussion

During 2004–2014, the 5-year survival from PC in PA was 82% (95% CI: 81.1–82.8%), with lower survival observed in the PSCI catchment area compared to the rest of PA. Within the PSCI catchment area, we found that PC survival rates were statistically significantly lower in rural Appalachian regions compared to urban Appalachian and urban non-Appalachian regions. Rural Appalachia was associated with lower PC survival compared to urban non-Appalachia, even after adjusting for sociodemographic and clinical factors. Various environmental and socioeconomic factors were also found to be associated with lower PC survival rates for these regions; thus, these factors may further explain the survival disparities observed between the PSCI catchment and non-catchment areas of the state, and among the urban or rural Appalachian/non-Appalachian regions specifically in the catchment area.

PC mortality rates in PA have been decreasing from 1990 (39.1 per 100,000 men) to 2017 (18.3 per 100,000 men) [33]. This decrease may be due to better and more rigorous treatment after diagnosis. However, there are populations who remain at higher risk for poor PC outcomes. Populations in rural and Appalachian areas are known to have poorer health outcomes overall compared to the rest of the U.S. [19, 34, 35]. In the PSCI catchment area, lower PC 3, 5, 10-year survival rates were observed in rural Appalachia compared to urban Appalachia and urban non-Appalachia. This finding is consistent with previous studies that found higher PC mortality rates and lower PC survival rates in rural Appalachia (and overall Appalachia) compared to non-Appalachia [7, 36]. In the present study, PC cases from rural Appalachia within the catchment area had more severe disease stage at diagnosis in terms of positive lymph nodes and distant metastasis compared to PC cases from urban Appalachia and non-Appalachia. These more advanced stages of disease at diagnosis may explain lower PC survival rates among rural Appalachian PC cases. In addition, Appalachian populations have been reported to have lower cancer screening rates compared to other geographical areas [37]; as a result, PC cases may present at more advanced stages of disease due to the lack of early detection resulting in poor PC outcomes. Based on a Medicare provider database assessment, as of September 2019 [38], the Penn State Health Hershey Medical Center is the only academic medical center with a cancer institute among the 135 hospitals identified within the PSCI catchment area. The Penn State Cancer Institute aims to make cancer screening and cancer treatment services more accessible to its 28-county catchment area so that cancer cases are identified earlier and are provided the appropriate treatment to improve cancer health outcomes.

Various reasons may contribute to the spatial disparities in PC survival that we observed in PA. We found that lower PC survival could be potentially associated with several health behavior and socio-economic risk factors (e.g., poor physical activity, diabetes, median household income) which are consistent with previous studies [39–42]. As for environmental factors, PA counties that had worse PC survivorship were areas that had higher levels of herbicide and insecticide usage, which are types of pesticides, and chemicals used for defoliation. In previous studies, positive associations between pesticide use and PC mortality have been found [43, 44]; however these study findings have been inconsistent and warrant further investigation. In addition, we found that the PA counties that had the lowest PC survival rates consequently had higher levels of several air pollutants that were listed in Table 4. Of these air pollutants, according to the International Agency for Research on Cancer, *ortho*-toluidine (o-toluidine) is classified as carcinogenic to human (Group 1). Dimethyl sulfate, benzyl chloride, epichlorohydrin, ethyl acrylate, and hydrazine are classified as probably carcinogenic to humans (Group 2A). Chloroprene, 2-nitropropane, antimony trioxide (this specific type is not specified in Table 4), hexachlorobenzene, nitrobenzene, and vinyl acetate are classified as possibly carcinogenic to humans (Group 2B) [45]*.* Unlike the other chemicals, antimony compounds have been linked to PC in which higher serum concentrations of antimony were associated with lower survival among PC patients after radical prostatectomy, suggesting its role in PC progression [46]. Based on a meta-analysis, hexachlorobenzene, a type of organochlorine pesticide, was found to be inversely associated (but not statistically significant) with PC risk in the general population [47]. Because the relationships between various environmental exposures, either through air or land, and PC remain unclear and not known, further examination through both epidemiologic and mechanistic studies are warranted for better understanding.

This study had several limitations. First, we used data from a single state due to the specific interest and mission of this research. However, the PCR has been recognized with gold (highest) award by the North American Central Cancer Registry (NACCR) for 24 years, of which 11 years of data were used for this study. Second, we lacked data on several important exposures (e.g., smoking, socio-economic status) at the individual-level, which is why this study utilized aggregate county-level data as a surrogate for further secondary assessment. Third, with regards to these aggregated risk factors (e.g., the CHR and EQI), one possible source for statistical bias is the modifiable areal unit problem, which may result from the shape and scale of aggregation units. Also, of note is that the existing community or health behavior-related factors are not specific to males aged 40 years old and above. Finally, some data were missing, limiting our access to complete case information such as inconsistent documentation of clinical factors (i.e., Gleason pattern/score) or PC treatment data (i.e., radiation therapy) and missing month/day information for cancer diagnosis date, among others.

Despite the limitations of the available data, this study had substantial strengths. As noted above, the PCR is a high-quality registry by the NAACCR, which we linked to most recent county-level CHR and EQI data, thus providing informative and comprehensive data resources for valid inference. We used AFT models as a more robust and informative approach than Cox proportional hazards models, considering geographical surrounds with a method more robust to departures from the proportional hazard assumption [28, 48, 49]. The findings can be used to inform targeted etiologic, epidemiologic, and health services research for investigators at the PSCI, to increase PC survival throughout its catchment area, especially in rural communities.

## Conclusions

In conclusion, we evaluated spatial patterns of prostate cancer mortality in PA, and found reduced survival from prostate cancer in the PSCI catchment area, especially in rural Appalachia. Future studies should examine the identified health community and environmental factors which may drive this reduced survival. Also, health care interventions to improve prostate cancer survival should be developed, implemented and evaluated in the PSCI catchment area.

## Supplementary information

**Additional file 1.** Supplmentary materials including statistical methods and additional analysis results.

## Data Availability

The datasets used for the current study are available based upon request to the corresponding author, Dr. Ming Wang, or directed to Jim Rubertone at the Bureau of Health Statistics & Registries, Pennsylvania Department of Health, Pennsylvania.

## Abbreviations

PC: Prostate Cancer
PA: Pennsylvania
PCR: Pennsylvania Cancer Registry
CHR: County Health Rankings
EPA: Environmental Protection Agency
PSCI: Penn State Cancer Institute
AFT: Accelerated Failure Times
CI: Confidence Interval/Credible Interval
SD: Standard Deviations
