# Supplementary material for: Spatial patterns in prostate Cancer-specific mortality in Pennsylvania using Pennsylvania Cancer registry data, 2004–2014
Source: BMC Cancer. 2020 May 6;20:394. doi: 10.1186/s12885-020-06902-5 (PMC7203834; doi:10.1186/s12885-020-06902-5)
Supplement: Supplementary file 1 — Additional file 1. Supplmentary materials including statistical methods and additional analysis results. [file 12885_2020_6902_MOESM1_ESM.docx]

**Supplementary Material for “Spatial Patterns in Prostate Cancer-specific mortality in Pennsylvania Using Pennsylvania Cancer Registry Data, 2004-2014”**

Ming Wang, PhD^1,2^; Emily Wasserman, MAS^1^; Nathaniel Geyer, DrPH^1^; Rachel M. Carroll, PhD^5^; Shanshan Zhao, PhD^6^; Lijun Zhang, PhD^2,4^; Raymond Hohl, MD, PhD^2,3,7^; Eugene J. Lengerich, MD^1,2,3^; Alicia C. McDonald, PhD^1,2^

^1^ Department of Public Health Sciences, Penn State College of Medicine, Hershey, PA, United States

^2^ Penn State Cancer Institute, Hershey, PA, United States

^3^ Penn State Milton S. Hershey Medical Center, Hershey, PA, United States

^4^ Penn State Institute of Personalized Medicine, Hershey, PA, United States

^5^ Department of Mathematics and Statistics, the University of North Carolina at Wilmington, NC, United States

^6^ Biostatistics and Computational Biology Branch, National Institute of Environmental Health Sciences, Research Triangle Park, NC, United States

^7^ Department of Pharmacology, Penn State College of Medicine, Hershey, PA, United States

Author e-mail addresses:

Ming Wang [muw22@psu.edu](mailto:muw22@psu.edu)

Emily Wasserman [ewasserman@phs.psu.edu](mailto:ewasserman@phs.psu.edu)

Nathaniel Geyer [NGeyer@phs.psu.edu](mailto:NGeyer@phs.psu.edu)

Rachel Carroll [drrmcarroll@gmail.com](mailto:drrmcarroll@gmail.com)

Shanshan Zhao [shanshan.zhao@nih.gov](mailto:shanshan.zhao@nih.gov)

Lijun Zhang [lzhang6@pennstatehealth.ps](mailto:lzhang6@pennstatehealth.ps)u.edu

Raymond Hohl [rhohl@pennstatehealth.psu.edu](mailto:rhohl@pennstatehealth.psu.edu)

Eugene J. Lengerich [ELengerich@phs.psu.edu](mailto:ELengerich@phs.psu.edu)

Alicia C. McDonald [amcdonald3@phs.psu.edu](mailto:amcdonald3@phs.psu.edu)

Correspondence concerning this article should be addressed to

Ming Wang, PhD

Associate Professor

Department of Public Health Sciences

Penn State College of Medicine and Cancer Institute

90 Hope Drive, Hershey, PA 17033

Email Contact: [muw22@psu.edu](mailto:muw22@psu.edu)

**A. Statistical Method:** Let $D_{ij}$denote the time to death due to PC from the date of diagnosis, and $C_{ij}$ to represent the corresponding censoring time for an individual $i$ in county $j (j=1,\ldots,67)$. Then ${t_{ij}=min(D}_{ij}, C_{ij})$is the observed survival time with the death indicator ${\delta_{ij}=I(D}_{ij}\leq C_{ij}).$ Also, $\boldsymbol{x}_{ij}$ is a vector of covariates, including individual-level risk factor from PCR (e.g., age at diagnosis, race, serum PSA, definitive treatment) as well as county-level variables from CHP and EQI databases. The AFT model treats the natural logarithm of survival time as the response variable, which is specified as

$$\log\left( D_{ij} \right)=\alpha_{0}+\boldsymbol{\beta}^{\boldsymbol{'}}\boldsymbol{x}_{ij}+w_{j}+\sigma\epsilon_{ij}$$

where $\alpha_{0}$ is the population level mean and $\sigma$ is a scale parameter which controls the variance of the survival curve. $\boldsymbol{\beta}$ is a vector of regression coefficients directly related to the logarithm of time to death due to PC, where a negative value indicates a decrease in survival time and a positive value indicates an increase in survival time. $\epsilon_{ij}$is the residual term which follows a distribution with the distribution function $F_{\epsilon}\left( \cdot\right).$ Here, we assume $\epsilon_{ij}$follows a standard normal distribution, thus $D_{ij}$ is lognormal distributed. The frailty term $w_{j}$ follows a conditional autoregressive distribution, accounting for spatial correlation among counties, where adjacent counties tend to be more correlated. The model goodness of fit is evaluated based on the deviance information criterion (DIC), while the Bayesian approach with the Gibbs sampler algorithm and non-informative prior distributions are utilized for parameter estimation and inference. Thus, the spatial frailty terms ($w_{j}'s$) are estimated for each county, showing spatial heterogeneity in survivorship (higher values indicate longer survival). We divide the counties into four survivorship categories based on quartiles of these spatial variation estimates. Then, a secondary assessment of spatial frailty estimates is conducted to explore the potential county-level risk factors $Y_{j}$ by taking the spatial correlation structure into account, which is shown below

$g \left( E(Y_{j}) \right)=\eta_{0}+\boldsymbol{\xi}^{\boldsymbol{'}}\boldsymbol{q}_{j}+\gamma_{j}$,

where $g$ is any known link function depending on the type of risk factors (e.g., an identity function for continuous variables, a logit function for binomial variables), $\boldsymbol{q}_{j}$ is the vector of survival risk quartiles based on spatial frailty estimates, and $\boldsymbol{\xi}$ are the corresponding parameters. Thus, the risk factors with significant differences between the first and fourth quartiles are detected. Those factors are considered to be potentially associated with PC spatial heterogeneity, represented by the spatial frailty in survival modeling.

**B. Additional analysis:**

**B.1.** Per reviewers’ suggestion, we conducted additional follow-up analysis evaluating the Kaplan-Meier (KM) curves stratified by the urban or rural Appalachia regions in Pennsylvania (the left panel of Figure 1 below) and comparing survival between the PSCI catchment versus non-catchment areas within rural-Appalachia (the right panel of Figure 1 below). The results show that there are statistically significant differences in survival among rural-Appalachia, urban-Appalachia, and urban-non Appalachia in Pennsylvania. In rural-Appalachia, the PSCI catchment area has a higher risk of mortality compared to the non-catchment area.

**B.2.** We conducted univariate analyses for initial variable screening, and also considered prior knowledge on potential risk factors in literature to explore our primary objective (spatial heterogeneity in prostate cancer mortality by the urban or rural Appalachia regions and the catchment area). Of note, we did not consider serum PSA when model fitting for two reasons. One reason is the collinearity issue due to potential correlations between serum PSA and other risk factors, such as stage of aggressiveness and lymph node status. There is a significantly high correlation between these factors based on correlation tests and ANOVA tests (*p*-values <0.001). The second reason is that there were about 12% cases missing serum PSA values (11,963 cases out of a total sample size of 94,274), which could lead to less power and potential convergence issue in particular under the Bayesian inference framework. Thus, due to these issues, we did not consider serum PSA in our modeling. With regards to the age variable (“1” if aged 60 years and above; otherwise “0”), we found that survival did not significantly differ by age group based on the log-rank test (*p*-value=0.1), thus, we did not include the age group in our modeling (see the KM curves in Figure 2 below). Furthermore, as a sensitivity analysis, we performed additional analysis by adding this age group variable in multivariate AFT model fitting, and the detected significant effects remained the same.


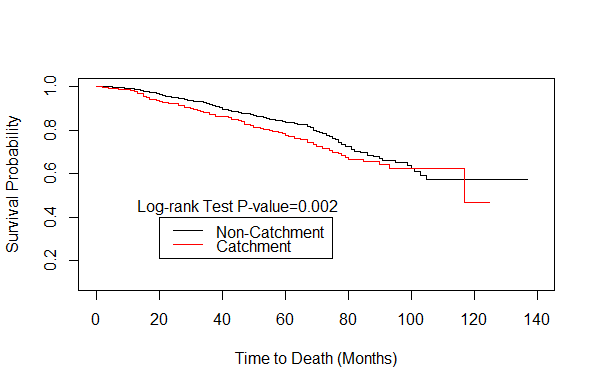

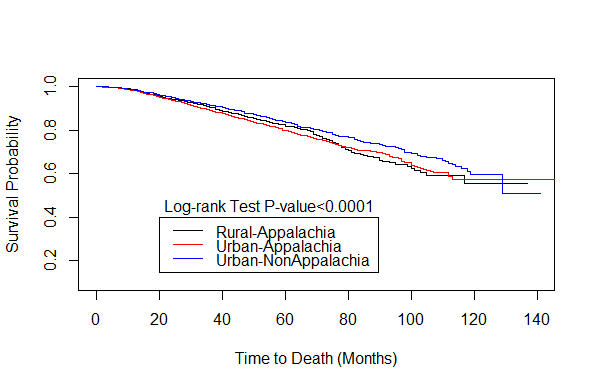


Figure 1. The KM-curves stratified by the urban or rural Appalachian regions for the whole Pennsylvania (the left panel) and by the PSCI catchment area for the rural-Appalachia area only (the right panel)


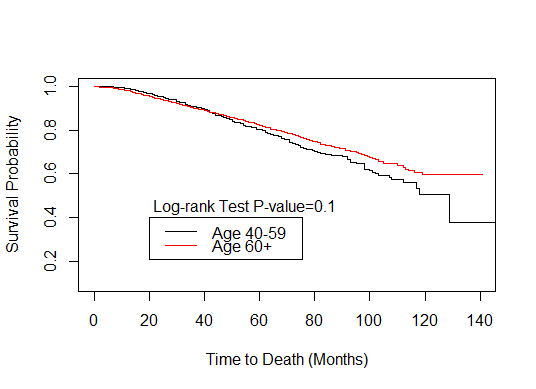


Figure 2. The KM-curves stratified by the age group for the whole Pennsylvania

**Supplement Table 1:** Summary statistics on the selected environmental risk factors based on secondary assessment of spatial frailties (1^st^ and 4^th^ quartile) from the AFT model in PA

|  | 1^st^ Quartile  Mean (SD) | 4^th^ Quartile  Mean (SD) |
| --- | --- | --- |
|  |  |  |
| **County Health Rankings** |  |  |
| *Poor physical health days/Physically Unhealthy Days (N)* | 3.63 (0.18) | 3.61 (0.25) |
| *Low birth weight (%)* | 7.81 (0.91) | 7.41 (0.87) |
| *Food Environment Index* | 8.15 (0.45) | 8.23 (0.37) |
| *Income inequality 80th Percentile* | 98,393 (14,204) | 124,068 (92,580) |
| *Number of Workers driving alone/long commute (N)* | 55,837 (48,415) | 3.61 (0.25) |
| *Premature Age-Adjusted Mortality* | 362.25 (52.39) | 7.41 (0.87) |
| *Diabetes prevalence/Diabetic (N)* | 11.5 (1.03) | 11.29 (1,.31) |
| *Median household income* | 53,226 (7,819) | 52,404 (12,582) |
| *Non-Hispanic White (N)* | 124,068 (92,580) | 182,629 (234,00) |
| **Land Domain** |  |  |
| *Percent defoliate chemical applied/total acres ¶* | 253.85 (703.23) | 61.15 (89.89) |
| *Herbicides (pounds)* | 32,826 (28,166) | 37,509 (38,062) |
| *Insecticides (pounds)* | 4,379 (3,825) | 5,182 (5,002) |
| **Air Domain** |  |  |
| *1,1,2-trichloroethane (tons emitted) ¶* | 338.36 (1349.28) | 146.58 (601.76) |
| *2,4-toluene diisocyanate (tons emitted) ¶* | 136.22 (161.37) | 73.27 (118.42) |
| *2-nitropropane (tons emitted) ¶* | 0.06 (0.16) | 0.01 (0.02) |
| *Acetonitrile (tons emitted) ¶* | 91.29 (217.14) | 893.57 (3,633.51) |
| *Acetophenone (tons emitted) ¶* | 6.27 (9.30) | 2.24 (2.41) |
| *Acrylic acid (tons emitted) ¶* | 7.99 (12.82) | 11.49 (28.93) |
| *Antimony compounds (tons emitted) ¶* | 7.09 (9.35) | 6.79 (11.64) |
| *Benzyl chloride (tons emitted) ¶* | 14.83 (51.65) | 2.27 (6.08) |
| *Bromoform (tons emitted) ¶* | 0.14 (0.17) | 0.05 (0.07) |
| *Chloroprene (tons emitted) ¶* | 1.12 (1.11) | 0.76 (0.96) |
| *Dibutylphthalate (tons emitted) ¶* | 45.35 (97.77) | 38.25 (128.83) |
| *Dimethyl phthalates (tons emitted) ¶* | 28.70 (75.72 | 9.62 (19.49) |
| *Dimethyl sulfate (tons emitted) ¶* | 0.22 (0.21) | 0.09 (0.10) |
| *Epichlorohydrin (tons emitted) ¶* | 0.33 (0.84) | 0.15 (0.25) |
| *Ethyl acrylate (tons emitted) ¶* | 14.72 (28.90) | 8.10 (20.24) |
| *Ethylidene dichloride (tons emitted) ¶* | 1.61 (2.17) | 2.81 (3.97) |
| *Hexachlorobenzene (tons emitted) ¶* | 0.69 (2.43) | 0.05 (1.09) |
| *Hexachlorobutadiene (tons emitted) ¶* | 0.02 (0.1) | 0.05 (0.03) |
| *Hydrazine (tons emitted) ¶* | 1.11 (2.41) | 0.01 (0.01) |
| *Isophorone (tons emitted) ¶* | 24.28 (40.42) | 12.81 (23.06) |
| *Methylhydrazine (tons emitted) ¶* | 0.49 (0.45) | 0.33 (0.27) |
| *Nitrobenzene (tons emitted) ¶* | 0.45 (1.08) | 0.20 (0.34) |
| *N,N-dimethylaniline (tons emitted) ¶* | 7.93 (4.70) | 5.88 (4.32) |
| *o-toluidine (tons emitted) ¶* | 0.03 (0.02) | 0.02 (0.02) |
| *Vinyl acetate (tons emitted) ¶* | 132.62 (367.96) | 34.27 (54.05) |

Note: *¶* Risk factor was quantified per 100,000 in the original scale; SD: standard deviation.
